# Germline sequencing in men with metastatic castration-resistant prostate cancer from the BARCODE2 study reveals a wide range of pathogenic variants in DNA repair genes

**DOI:** 10.1038/s44276-023-00024-8

**Published:** 2024-02-15

**Authors:** Sarah Benafif, Ann-Britt Jones, Susan Merson, Reshma Rageevakumar, Eva McGrowder, Matthew Tyler, Fay Cafferty, Matthew Hogben, Nafisa Hussain, Elizabeth Bancroft, Alison Reid, Sarah Wakerell, Questa Karlsson, Edward Saunders, Ian Whitmore, Karina Dalsgaard Sorensen, Nening Dennis, Evie Black, Angela Wood, Kate Richards, Kathryn Lees, Carla Perna, Alison Falconer, Jamie Mills, Robert Hughes, Shiyam Kumar, Christos Mikropoulos, Stephanie Burnett, Gerhardt Attard, Emma Hall, Zsofia Kote-Jarai, Ros Eeles

**Affiliations:** 1 The Institute of Cancer Research, Sutton, UK; 2 The Royal Marsden NHS Foundation Trust, London, UK; 3 University College London, London, UK; 4 Aarhus University Hospital, Aarhus, Denmark; 5 Maidstone and Turnbridge Wells NHS Trust, London, UK; 6 Royal Surrey NHS Foundation Trust, London, UK; 7 Imperial College Healthcare NHS Trust, London, UK; 8 Nottingham University Hospitals NHS Trust, Nottingham, UK; 9 East and North Hertfordshire NHS Trust, Stevenage, UK; 10 Yeovil District Hospital NHS Foundation Trust, Yeovil, UK; 11 Medway NHS Foundation Trust, Gillingham, UK

## Abstract

**Background:**

The presence of germline mutations plays an increasingly important role in risk assessment and treatment of prostate cancer (PrCa). Screening for high-risk mutations in subsets of patients is becoming routine. We explore the prevalence of germline genetic mutations in men with metastatic castration-resistant prostate cancer (mCRPC) recruited to the BARCODE2 trial.

**Methods:**

The BARCODE2 trial is a two-part study investigating the response to carboplatin chemotherapy in mCRPC patients carrying a germline variant in a DNA repair gene (DRG). We report interim data from Part 1, in which participants are recruited for germline genetic testing using a customised next-generation sequencing panel consisting of 115 genes.

**Results:**

These interim results (*N* = 220) demonstrate a similar frequency of germline DRG variants in mCRPC patients compared with previously published data (15% detection rate). No significant clinical differences were identified between all carriers and non-carriers, though *BRCA2/ATM* carriers were found to have a shorter time to mCRPC diagnosis.

**Conclusions:**

Germline pathogenic/likely pathogenic (P/LP) variants in *BRCA2 and ATM* genes are associated with a shorter time to progression and rarer P/LP variants in other DRG genes may play a role in mCRPC. This justifies the use of routine screening of men with advanced PrCa for germline variants and supports the need for an expanded panel test.

## Introduction

Inherited mutations in certain genes increase the risk of prostate cancer (PrCa) development and, for some genes, e.g., *BRCA2*, are associated with aggressive disease and poorer outcomes. This has led to calls for routine germline genetic screening in men diagnosed with metastatic PrCa or those with localised disease at a young age and/or with a strong family history [National Comprehensive Cancer Network (NCCN) Prostate Cancer Guidelines 2.2023]. Germline variants in DNA repair genes (DRG) appear to be associated with higher risk features such as nodal and/or distant metastatic disease at diagnosis [1–3]. The reported frequency of germline variants in DRG varies from 12 to 19% in patients with metastatic PrCa [4]. The most frequently altered germline DRG genes are *BRCA2* and *ATM* [2]. The presence of variants in these genes may also impact active surveillance pathways used for the management of low-grade localised PrCa [5]. In addition, several studies have reported an increased incidence of PrCa in men with Lynch Syndrome [6, 7]. Detecting these mutations can also impact personalised treatment as checkpoint inhibitors for tumours with mismatch repair deficiency have been approved regardless of primary tumour type by the U.S. Food and Drug Administration (FDA) and are under review by The National Institute for Health and Care Excellence (NICE) in the UK.

Consequently, targeted therapies are under investigation in this setting for the treatment of PrCa. Studies have shown the efficacy of poly(adenosine diphosphate–ribose) polymerase inhibitors (PARPi) in patients with germline or somatic mutations in homologous recombination repair (HR) genes [8–10]. Olaparib was approved by the U.S. Food and Drug Administration (FDA) in 2020 for mCRPC associated with mutations in HR genes and was recently approved for use in men with *BRCA*-mutated metastatic PrCa in the UK (https://www.fda.gov/drugs/resources-information-approved-drugs/fda-approves-olaparib-hrr-gene-mutated-metastatic-castration-resistant-prostate-cancer). Retrospective case studies have shown platinum chemotherapy has a durable response in *BRCA2* mutation carriers with PrCa [11, 12].

The BARCODE2 trial (NCT02955082) is the first prospective trial to investigate the presence of a germline genetic mutation in a set of DRG (Part 1) and the efficacy of carboplatin (Part 2) in men with mCRPC carrying a germline P/LP variant. This is a unique opportunity to assess whether this cohort demonstrates evidence of poorer prognostic features as seen with carriers of *BRCA2* and *ATM* variants.

Here, we present the results of germline sequencing for the first 220 patients enrolled in Part 1 of BARCODE2 with the aim of estimating the prevalence of DRG mutations in mCRPC and describing associated disease characteristics.

## Materials and methods

### Patient population

The BARCODE2 trial is an ongoing, two-part study recruiting patients via uro-oncology clinics at The Royal Marsden NHS Foundation Trust (RMH) and collaborating centres. Part 1 of the trial screens for germline genetic alterations in a customised panel of 115 DRG. To enter, all patients were required to have a diagnosis of prostate adenocarcinoma with castrate levels of testosterone and evidence of disease progression after at least one of the following: docetaxel, abiraterone, or enzalutamide. Study participants found to have a variant in a DNA repair gene meeting study criteria for P/LP classification are then offered treatment with carboplatin chemotherapy (Part 2).

Exclusion criteria include previous treatment with carboplatin and/ or a PARPi. Informed consent was obtained, and patients provided a blood sample for DNA extraction and sequencing. Demographic and clinical data were collected for all participants.

Data from Part 2 of the trial is not presented here as recruitment continues. The study allows direct entry to Part 2 if a patient is known to carry an actionable germline mutation in a DRG. These patients are excluded from the current analysis since they did not undergo sequencing within the study.

### Sequencing platform and variant calling

Germline DNA extracted from whole blood was sequenced utilising a study-specific next-generation sequencing (NGS) gene panel. The Agilent SureSelect XT Custom 0.5–2.9 Mb bait capture library was used to design RNA sequences (baits) to target the exons of 115 genes (Supplementary Table 1). The capture baits were designed to include 50 base pairs on either side of each exon to allow the sequencing of splice regions. Library preparation was carried out using the Agilent SureSelect^QXT^ Target Enrichment System prior to sequencing on an Illumina MiSeq machine.

De-multiplexed and adaptor-trimmed MiSeq-generated FASTQ files were processed and analysed using the SureCall program (Version 4.0, Agilent Technologies). This is an integrated package that performs alignment of reads to the reference genome (GRCh37 release, hg19, February 2009), removal of duplicates, variant calling (SNPPET Caller) and variant annotation. SureCall FASTQ processing and variant calling was validated using the Genome Analysis Toolkit (GATK version 3.5) pipeline. Target coverage was 20× or higher for at least 80% of target bases. Sanger sequencing was used to confirm all germline variants that were deemed actionable within the trial.

The primary aim was to identify germline pathogenic and likely pathogenic (P/LP) variants that would qualify patients for entry into the treatment part of the trial (Part 2). Variants were considered P/LP based on the following criteria: nonsense, frameshift or canonical ±1.2 splice site variants predicted to cause protein truncation or nonsense-mediated decay, with an allele frequency ≤0.5% among any population using gnomAD database, and/ or reported as P/LP in ClinVar. Variants occurring in the final exon of a gene were excluded, unless known to be clinically pathogenic. Protein-truncating variants called by SureCall were reviewed using the software’s integrated genome viewer. Additional annotation, including population frequency data from ExAC [13] and gnomADv2.1.1 [14] plus clinical data from ClinVar [15], were used to aid variant interpretation and classification. The American College of Medical Genetics guidelines for interpreting genomic variants were followed [16].

### Statistical analysis

Clinical and demographic characteristics of the cohort are described using standard summary statistics, and the prevalence of germline P/LP variants is estimated as a proportion of patients screened. Differences in baseline PrCa staging and family or personal history of cancer were assessed using the *χ*^2^ test or, in the case of categories with small numbers, Fisher’s exact test. Age at PrCa diagnosis, age at CRPC diagnosis, interval to CRPC and PSA at diagnosis were compared using a *t*-test for two independent samples if the normality assumption held or, otherwise, the Mann–Whitney two-sample test. A Kaplan–Meier plot (and associated log-rank test) was also used to compare time to CRPC onset from initial diagnosis. Ethnicity was not compared due to limited variation.

Since variants in the *BRCA2* and *ATM* genes have been previously shown to be associated with worse PrCa outcomes, the above analysis was repeated considering patients with P/LP variants in these genes compared to those patients with no actionable variant.

All statistical tests were conducted at a significance threshold of 5%. Comparisons of clinical characteristics were exploratory and not powered to detect differences between the groups.

## Results

### Baseline characteristics

A total of 230 study participants were recruited between May 2017 and January 2023. Two participants were excluded from all analyses since these entered directly into Part 2 of the trial and their germline status was already known and did not require on-study germline sequencing. The median age of 228 participants was 68 years (range 41–84) at study entry. Most participants (88.6%) were of European ancestry (Supplementary Table 2).

Of the 228 participants who underwent germline sequencing, 4 patients were excluded from analyses due to the identification of a variant in *POLQ*, which was deemed to be of uncertain significance within the context of the study. In addition, four patients were found to carry a pathogenic variant in *MUTYH*, which was deemed not actionable for Part 2 of the study and subsequently excluded (Supplementary Material). Thus, 220 patients were included in the statistical analysis, of which 34 (15.5%, 95% CI [10.9–20.9%]) were found to carry at least one actionable germline P/LP variant (Supplementary Table 3), with actionability defined as suitable for carboplatin treatment within Part 2 of the study. Of these patients, eight had a P/LP in *BRCA2* and two in *ATM*; hence, the prevalence of *BRCA2/ATM* carriers was 4.5% (95% CI 2.2–8.2%) and this subgroup was analysed separately.

**Fig. 1:**
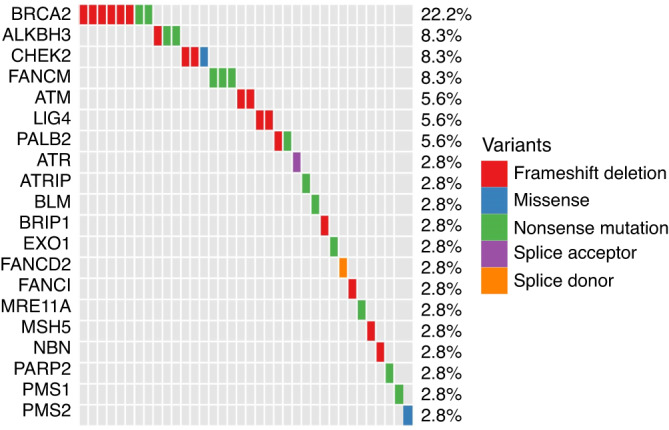
Pathogenic/likely pathogenic variants identified in 220 analysed samples.

The most frequently altered gene was *BRCA2* (8 P/LP were identified in seven participants) followed by *ALKBH3* (*n* = 3). Figure 1 lists the genes with identified P/LP variants (see Supplementary Material for full gene/variant details). Not all variants were actionable, and some participants had two variants identified.

Table 1 shows the PrCa clinical characteristics at diagnosis and family history of cancer for the carrier and non-carrier groups. Carriers tended to have higher Gleason scores (8–10) and PSA at diagnosis and were more likely to have nodal involvement and/ or metastatic disease at diagnosis, though these differences weren’t statistically significant. A larger proportion of carriers had known cases of cancer in any relative, cancer in a first-degree relative, and breast or ovarian cancer in a first-degree relative.

**Table 1:** Baseline characteristics and time to progression in carriers and non-carriers

	Carriers	Non-carriers	*P* value^a^
*n* (%)	34 (15.4)	186 (84.5)	
Gleason score at diagnosis (total score, *n* = 197)			
4–6	1 (3.4%)	9 (5.4%)	0.509
7	7 (24.1%)	59 (35.1%)	
8–10	21 (72.4%)	100 (59.5%)	
Lymph node stage at diagnosis (*n* = 180)			
N0	14 (50.0%)	71 (46.7%)	0.344
N1 or N2^b^	14 (50.0%)	68 (44.7%)	
NX	0 (0.0%)	13 (8.6%)	
Metastatic disease at diagnosis (*n* = 220)			
Yes	22 (64.7%)	97 (52.2%)	0.195
No	12 (35.3%)	89 (47.8%)	
Previous malignancy (*n* = 220)			
Yes	3 (8.8%)	22 (11.8%)	0.612
No	31 (91.2%)	164 (88.2%)	
Family history of cancer (*n* = 212)			
Any cancer (any relative)	28 (84.8%)	138 (77.1%)	0.321
Prostate cancer (any relative)	8 (24.2%)	61 (34.1%)	0.268
Prostate cancer in first-degree relative	7 (21.2%)	42 (23.5%)	0.778
Breast or ovarian cancer in first-degree relative	8 (24.2%)	32 (17.9%)	0.391
Any cancer in first-degree relative	25 (75.8%)	119 (66.5%)	0.294
Prostate, breast, or ovarian cancer in first-degree relative	12 (34.6%)	67 (37.4%)	0.907
	Median [range]	Median [range]	*P* value^c^
PSA at diagnosis (*n* = 211)	50.0 [3, 5000]	38.4 [2, 7700]	0.659
Age at initial prostate cancer diagnosis	60.0 [39, 77]	61.1 [43, 81]	0.147
Age at diagnosis of castration-resistant disease (*n* = 220)	65.0 [40, 81]	66. 9 [44, 83]	0.086
Months from initial diagnosis to CRPC onset (*n* = 220)	29.3 [1, 153]	38.2 [6, 262]	0.407

^a^*χ*^2^ test or Fisher’s exact test to compare proportions between carriers and non-carriers.

^b^Patients with grades N1 or N2 were grouped for the purpose of this analysis.

^c^t-test for two independent samples or Mann–Whitney two-sample test in the case of non-normally distributed data (used for age at CRPC onset and interval between initial diagnoses and CRPC onset).

**Fig. 2:**
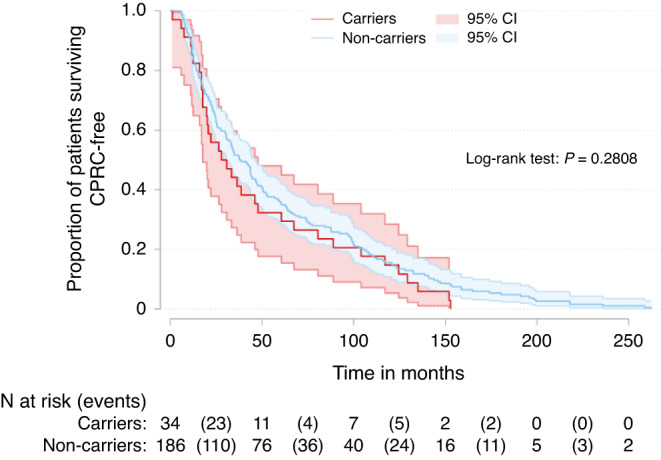
Time to CRPC onset from initial diagnosis of prostate cancer stratified by carrier status.

Carriers were slightly younger at diagnosis (65 vs 66.9 years, median) and had shorter time between initial PrCa diagnosis and CRPC diagnosis, 29.3 vs 38.2 months median respectively; however, these differences were not statistically significant. The Kaplan–Meier graph in Fig. 2 compares the time to CRPC from initial diagnosis in carriers vs non-carriers showing no significant difference between groups.

In the subgroup analysis of *BRCA2/ATM* carriers vs non-carriers, we found age at initial PrCa diagnosis and at CRPC diagnosis was lower among *BRCA2/ATM* carriers with a shorter interval to CRPC onset, 24.3 vs 38.2 months, respectively. Comparison of disease characteristics and time to progression is summarised in Table 2. *BRCA2/ATM* carriers were found to have more aggressive features of PrCa at diagnosis: Gleason score 8–10 (88.9% vs 59.5%), metastatic disease at diagnosis (80% vs 52.2%) and higher median PSA at diagnosis (59.0 vs 38.4). These differences did not attain statistical significance.

**Table 2:** Baseline characteristics and time to progression in *BRCA2/ATM* carriers and non-carriers

	*BRCA2/ATM* carriers	Non-carriers	*P* value^a^
*n* (%)	10 (5.1)	186 (94.9)	
Gleason score at diagnosis (total score, *n* = 177)			
4–6	0 (0.0%)	9 (5.4%)	0.250
7	1 (11.1%)	59 (35.1%)	
8–10	8 (88.9%)	100 (59.5%)	
Lymph node stage at diagnosis (*n* = 158)			
N0	2 (33.3%)	71 (46.7%)	0.669
N1 or N2^b^	4 (66.7%)	68 (44.7%)	
NX	0 (0.0%)	13 (8.6%)	
Metastatic disease at diagnosis (*n* = 196)			
Yes	8 (80.0%)	97 (52.2%)	0.109
No	2 (20.0%)	89 (47.8%)	
Previous malignancy (*n* = 196)			
Yes	0 (0.0%)	22 (11.8%)	0.607
No	10 (100.0%)	164 (88.2%)	
Family history of cancer (*n* = 189)			
Any cancer (any relative)	8 (80.0%)	138 (77.1%)	>0.999
Prostate cancer (any relative)	1 (10.0%)	61 (34.1%)	0.170
Prostate cancer in first-degree relative	1 (10.0%)	42 (23.5%)	0.460
Breast or ovarian cancer in first-degree relative	2 (20.0%)	32 (17.9%)	>0.999
Any cancer in first-degree relative	6 (60.0%)	119 (66.5%)	0.736
Prostate, breast, or ovarian cancer in first-degree relative	3 (30.0%)	67 (37.4%)	0.747
	Median [range]	Median [range]	*P* value^c^
PSA at diagnosis (*n* = 188)	59.0 [5, 400]	38.4 [2, 7700]	0.694
Age at initial prostate cancer diagnosis (*n* = 196)	59.7 [39, 72]	61.1 [43, 81]	0.205
Age at diagnosis of castration-resistant disease (*n* = 196)	64.4 [40, 73]	66.9 [44, 83]	0.084
Months from initial diagnosis to CRPC onset (*n* = 196)	24.3 [6, 89]	38.2 [6, 262]	0.120

^a^*χ*^2^ test or Fisher’s exact test (in the case of low cell counts).

^b^Patients with grades N1 or N2 were grouped for the purpose of this analysis.

^c^*t*-test for two independent samples or Mann–Whitney two-sample test in the case of non-normally distributed data (used for age at CRPC onset and interval between initial diagnoses and CRPC onset).

**Fig. 3:**
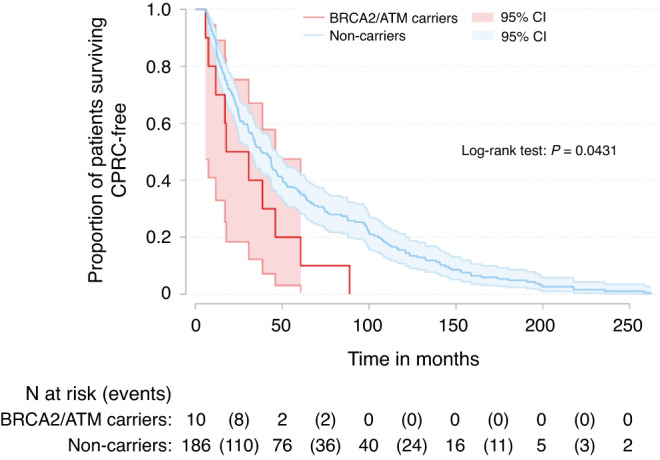
Time to CRPC onset from initial diagnosis of prostate cancer in *BRCA2/ATM* carriers and non-carriers.

Figure 3 depicts the time between PrCa diagnosis and CRPC onset in the *BRCA2/ATM* carriers and non-carriers. The interval to CRPC onset was significantly shorter in *BRCA2/ATM* carriers (log-rank test *P* = 0.043).

## Discussion

It is widely accepted that germline variants in certain genes such as *BRCA2* and *ATM* confer an elevated risk of developing PrCa [17]. The carrier rates of germline mutations in mCRPC in published datasets vary depending on the number of genes included in a study panel. We designed a trial-specific NGS panel to include 115 DNA repair genes, known cancer predisposition and candidate genes. This interim analysis of 220 mCRPC patients prospectively recruited in the UK, found that 15% had a P/LP variant. This is similar to the carrier frequency reported elsewhere [18] .

In our dataset, carriers tended to have features of more aggressive disease compared with non-carriers, although the differences were not statistically significant. It is noted that the group of carriers was relatively small meaning that statistical power for comparisons was limited.

Several studies have confirmed an association between germline *BRCA2/ATM* variants and a high-risk PrCa phenotype [2, 19]. A sub-analysis of *BRCA2/ATM* carriers in our cohort suggested a younger age at CRPC diagnosis (*P* = 0.08) and a shorter interval between initial PC diagnosis and CRPC onset (*P* = 0.04) compared with non-carriers, in keeping with our previous findings of BRCA association with aggressive PrCa [1].

Like the initial analysis of all carriers and non-carriers, the sub-analysis of *BRCA2/ ATM* carriers was limited by low statistical power making it difficult to isolate meaningful differences between subgroups.

The BARCODE2 trial utilises a bespoke study-specific gene panel to assess 115 germline genes. These were selected based on published data related to the association of PrCa with germline variants in DNA repair genes [4]. The remainder of the genes included were selected based on our team’s previously reported data in this setting [20, 21].

When grouped according to specific DNA repair pathways, homologous recombination (HR) genes made up approximately 75% of all carriers. It is unclear if all HR gene mutation carriers may respond to carboplatin or PARPi in a similar manner to BRCA and *ATM* carriers and studies to investigate this are underway [17]. In keeping with previous reports, the most altered germline gene in BARCODE2 was *BRCA2* (8 of 34 (23.5%) carriers). By sequencing many genes, we identified variants in 20 DRG not previously investigated. The second most frequently altered genes are those involved in interstrand crosslink repair (ICL). Five cases were carriers of a variant within the *FANC* gene family (in *FANCD2, FANCI* and *FANCM)*, which is 14.7% of all the carriers. The genes involved in the Fanconi Anaemia (FA) pathway function in maintaining genomic stability. Beyond *BRCA1/2*, some monoallelic germline variants within the FA family may increase cancer susceptibility [22]. One *FANCM* variant was detected in three of our cases and as this variant produces truncated proteins lacking C-binding domains resulting in loss of functional HR, this is likely behind the association with impaired DNA repair in advanced PrCa. We also identified *CHEK2* variants in three cases; two of these were 1100delC mutations, which have been linked with PrCa predisposition, and one missense variant also reported to be associated with elevated PrCa risk. These variants were identified in participants where another actionable P/LP variant was also found.

Variants in *ALKBH3* were detected in 3 cases (3 of 34, 8.8% of carriers). It encodes a protein involved in the repair of DNA damaged by alkylation and preferentially targets single-stranded DNA direct reversal repair. *ALKBH3* has been implicated in PrCa development as it is overexpressed in PrCa cells but not in benign prostatic hyperplasia or in normal prostate epithelium [23]. These findings have suggested that *ALKBH3* could be targeted for treatment and preclinical data have already been reported [24, 25]. Whether germline *ALKBH3* variants will confer sensitivity to platinum chemotherapy in PrCa remains to be seen.

Our study supports previous findings that a wider DRG panel should be tested, particularly in advanced PrCa cases as this may lead to alternative treatment strategies but could also allow cascade testing to family members who in turn could be managed with screening strategies before PrCa development. Our study identified one germline P/LP Lynch Syndrome variant (*PMS2*). A recent international prospective PrCa screening study showed that carriers of *MSH2* and *MSH6* mutations develop more aggressive PrCa compared to age-matched controls [26]. Targeted screening programmes among unaffected men with germline variants, a family history of PrCa or men of black African, Caribbean descent are under investigation (e.g., the IMPACT [27] and PROFILE [NCT02543905] studies [28]).

We acknowledge several limitations within this initial analysis of the BARCODE2 NGS findings. The study population lacks ethnic diversity as it consists of predominantly White individuals. The number of cases sequenced to date is relatively small and hence we have low statistical power. Recruitment to the trial is ongoing and sample size for the evaluation of efficacy (Part 2) in the gene subgroups was calculated based on population estimates available at the time of protocol finalisation. Once complete, re-analysis of carrier vs non-carrier characteristics will be performed as well as reporting of response rates to carboplatin.

PrCa progression intervals were documented prior to study entry and captured by the study team after trial entry. Hence, enrolled patients had not followed a common assessment schedule since PrCa diagnosis to ensure consistent documentation of time to castration resistance.

This study does not include prospective tumour DNA sequencing and therefore investigation of the impact of germline variants in novel genes such as *ALKBH3* has not been possible so far. Tumour DNA analysis to identify loss of heterozygosity or a ‘second hit’ alteration in a gene affected in the germline would allow an in-depth study of a potential role in PrCa development or progression. Plasma samples for circulating tumour DNA (ctDNA) analyses are being collected for future investigation.

In the UK, germline testing has recently been approved by the National Health Service (NHS) for PrCa patients meeting specific criteria and includes *BRCA1/2, ATM, PALB2, CHEK2, MLH1* and *MSH2/6*. As research continues in this setting with varied gene sets under investigation, the aim is to identify all the relevant genes to include in PrCa NGS panels that will guide screening and treatment options. This study shows further research is warranted to help identify the prevalence and impact of lesser recognised DRG variants among patients with metastatic PrCa.

## Supplementary Information

Supplementary material

## Data Availability

The overall analysis results are in the paper and the data used are shown in the tables and supplementary material.
