# Supplementary material for: Germline sequencing in men with metastatic castration-resistant prostate cancer from the BARCODE2 study reveals a wide range of pathogenic variants in DNA repair genes
Source: BJC Rep. 2024 Feb 15;2:15. doi: 10.1038/s44276-023-00024-8 (PMC11507020; doi:10.1038/s44276-023-00024-8)
Supplement: Supplementary file 1 — Supplementary material [file 44276_2023_24_MOESM1_ESM.docx]

**Supplementary material**

**Supplementary Table 1.**

**BARCODE2 Gene Panel n=115**

| **BARCODE2 Gene Panel** | | | | |
| --- | --- | --- | --- | --- |
| *ALKBH3* | *EME1* | *MLH1* | *POLD1* | *RNASEL* |
| *ANO7* | *EME2* | *MLH3* | *POLE* | *RPA1* |
| *APEX1* | *ERCC2* | *MMS19* | *POLK* | *SETMAR* |
| *AR* | *ERCC5* | *MNAT1* | *POLM* | *SLX4* |
| *ATM* | *ERCC6* | *MPG* | *POLN* | *SMAD4* |
| *ATR* | *ESR2* | *MRE11A* | *POLQ* | *SMARCA4* |
| *ATRIP* | *EXO1* | *MSH2* | *POT1* | *SMUG1* |
| *BAP1* | *FAM175A* | *MSH5* | *PRSS1* | *SPOP* |
| *BARD1* | *FANCA* | *MSH6* | *PTCH1* | *STK11* |
| *BLM* | *FANCD2* | *MSR1* | *PTEN* | *TDG* |
| *BRCA1* | *FANCI* | *MUTYH* | *RAD1* | *TOP2A* |
| *BRCA2* | *FANCL* | *NABP2* | *RAD50* | *TOP2B* |
| *BRIP1* | *FANCM* | *NBN* | *RAD51B* | *TOP3A* |
| *CCNH* | *GADD45A* | *NEIL1* | *RAD51C* | *TP53* |
| *CDC25C* | *GEN1* | *NEIL2* | *RAD51D* | *TP53BP1* |
| *CDH1* | *GTF2H2* | *NTHL1* | *RAD52* | *WRN* |
| *CDK4* | *GTF2H3* | *OGG1* | *RAD54B* | *XAB2* |
| *CDKN2A* | *GTF2H4* | *PALB2* | *RAD54L* | *XPA* |
| *CHD1* | *HOXB13* | *PARP2* | *RB1* | *XPC* |
| *CHEK1* | *HUS1* | *PER1* | *RECQL* | *XRCC1* |
| *CHEK2* | *LIG1* | *PMS1* | *RECQL4* | *XRCC2* |
| *CLK2* | *LIG3* | *PMS2* | *RECQL5* | *XRCC4* |
| *DCLRE1A* | *LIG4* | *PNKP* | *RINT1* | *XRCC5* |

|  | **Frequency** | **Percent** |
| --- | --- | --- |
| **Ethnicity (n=228)** |  |  |
| White European | 202 | 88.6% |
| Black (African or Caribbean) | 11 | 4.8% |
| Asian (South-East Asian or Middle Eastern) | 11 | 4.8% |
| Mixed | 3 | 1.3% |
| Other | 1* | 0.4% |
|  |  |  |
| **Family history (n=220)** |  |  |
| Any cancer (any relative) | 171 | 77.7% |
| Prostate cancer (any relative) | 70 | 31.8% |
| Prostate cancer in first degree relative | 50 | 22.7% |
| Breast or ovarian cancer in first degree relative | 42 | 19.1% |
| Any cancer in first degree relative | 149 | 67.7% |
| Prostate, breast or ovarian cancer in first degree relative | 81 | 36.8% |

| Supplementary Table 2. Ethnicity and Family History of Cancer  *White Canadian    Supplementary Material **Clinical characteristics of excluded patients** A recurrent *POLQ* variant was identified in six (2.6%) patients: c.4262_4268delTACTATT (rs546221341). This frameshift variant in exon 16 is predicted to be deleterious (CADD score of 26). As the gnomAD population frequency of this allele is 0.0057 (0.0078 in the European non-Finnish population) and above 0.5%, it was not deemed actionable within the trial and was classed as a variant of unknown significance (VUS). Among the six carriers, two individuals also carried a pathogenic variant in another DNA repair gene: *ATM* and *BRCA2*. In summary, four patients were excluded due to the *POLQ* variant alone and another four patients were excluded due to a heterozygous *MUTYH* mutation.  Supplementary Table 3    Pathogenic and likely pathogenic germline variants identified in BARCODE2. | | | |
| --- | --- | --- | --- |
| Gene | **Variant** | **Effect** | **Frequency** |
| *ALKBH3* | c.208C>T; p.Arg70Ter | Nonsense | 1 |
| *ALKBH3* | c.368_369del; p.Glu123GlyfsTer43 | Frameshift | 1 |
| *ALKBH3* | c.381T>G; p.Tyr127Ter | Nonsense | 1 |
| *ATM* | c.3292del; p.Gln1098ArgfsTer11 | Frameshift | 1 |
| *ATM* | c.3802del; p.Val1268Ter | Frameshift | 1 |
| *ATR* | c.2634-1G>A | Splice | 1 |
| *ATRIP* | c.955C>T; p.Gln319Ter | Nonsense | 1 |
| *BLM* | c.2695C>T; p.Arg899Ter | Nonsense | 1 |
| *BRCA2* | c.2834_2835del; p.Lys945ArgfsTer13 | Frameshift | 1 |
| *BRCA2* | c.3847_3848del; p.Val1283LysfsTer2 | Frameshift | 1 |
| *BRCA2* | c.4914_4915del; p.Lys1638AsnfsTer3 | Frameshift | 1 |
| *BRCA2* | c.5217T>A; p.Tyr1739Ter | Nonsense | 1 |
| *BRCA2* | c.5946del; p.Ser1982ArgfsTer22 | Frameshift | 2 |
| *BRCA2* | c.7360del; p.Ile2454PhefsTer13 | Frameshift | 1 |
| *BRCA2* | c.9599C>G; p.Ser3200Ter | Nonsense | 1 |
| *BRIP1* | c.2992_2995del; p.Lys998GlufsTer60 | Frameshift | 1 |
| *CHEK2* | c.470T>C; p.Ile157Thr | Missense | 1 |
| *CHEK2* | c.1100del; p.Thr367MetfsTer15 | Frameshift | 2 |
| *EXO1* | c.1241C>A; p.Ser414Ter | Nonsense | 1 |
| *FANCD2* | c.2715+1G>A | Splice | 1 |
| *FANCI* | c.912_916del; p.Asn304LysfsTer23 | Frameshift | 1 |
| *FANCM* | c.5791C>T; p.Arg1931Ter | Nonsense | 3 |
| *LIG4* | c.613del; p.Ser205LeufsTer29 | Frameshift | 1 |
| *LIG4* | c.1271_1275del; p.Lys424ArgfsTer20 | Frameshift | 1 |
| *MRE11A* | c.1090C>T; p.Arg364Ter | Nonsense | 1 |
| *MSH5* | c.1747del; p.Asp583ThrfsTer23 | Frameshift | 1 |
| *NBN* | c.657_661del; p.Lys219AsnfsTer16 | Frameshift | 1 |
| *PALB2* | c.1547del; p.Arg516LysfsTer45 | Frameshift | 1 |
| *PALB2* | c.3113G>A; p.Trp1038Ter | Nonsense | 1 |
| *PARP2* | c.1480C>T; p.Gln481Ter | Nonsense | 1 |
| *PMS1* | c.1888C>T; p.Arg630Ter | Nonsense | 1 |
| *PMS2* | c.137G>T; p.Ser46Ile | Missense | 1 |
